# Immunoglobulin Replacement Therapy Versus Antibiotic Prophylaxis as Treatment for Incomplete Primary Antibody Deficiency

**DOI:** 10.1007/s10875-020-00841-3

**Published:** 2020-11-18

**Authors:** Bas M. Smits, Ilona Kleine Budde, Esther de Vries, Ineke J. M. ten Berge, Robbert G. M. Bredius, Marcel van Deuren, Jaap T. van Dissel, Pauline M. Ellerbroek, Michiel van der Flier, P. Martin van Hagen, Chris Nieuwhof, Bram Rutgers, Lieke E. A. M. Sanders, Anna Simon, Taco W. Kuijpers, Joris M. van Montfrans

**Affiliations:** 1 Department of Pediatric Immunology and Infectious Diseases, UMC Utrecht, Lundlaan 6, 3584 EA Utrecht, The Netherlands; 2 Clinical Operations, Sanquin Plasma Products B.V, Amsterdam, The Netherlands; 3 Department of Tranzo, Tilburg School of Social and Behavioral Sciences, Tilburg University, Tilburg, The Netherlands; 4 Department of Jeroen Bosch Academy Research, Jeroen Bosch Hospital, ‘s-Hertogenbosch, The Netherlands; 5 Department of Internal Medicine, Amsterdam University Medical Centers, Academic Medical Center, Amsterdam, The Netherlands; 6 Department of Pediatrics, Leiden University Medical Center, Leiden, The Netherlands; 7 Department of Internal Medicine, Radboud UMC, Nijmegen, The Netherlands; 8 Department of Infectious Diseases, Leiden University Medical Centre, University of Leiden, Leiden, The Netherlands; 9 Division of Internal Medicine and Dermatology, Department of Infectious Diseases, University Medical Center Utrecht, Utrecht, The Netherlands; 10 Pediatric Infectious Diseases and Immunology, Radboudumc Amalia Children’s Hospital, Nijmegen, The Netherlands; 11 Department of Internal Medicine/Immunology, Erasmus University Medical Centre, Rotterdam, The Netherlands; 12 Department of Allergology and Clinical Immunology, Maastricht University Medical Centre (MUMC+), Maastricht, The Netherlands; 13 Department of Rheumatology and Clinical Immunology, University Medical Center Groningen, University of Groningen, Groningen, The Netherlands; 14 Department of Paediatric Immunology and Infectious Diseases, Emma Children’s Hospital, AUMC, University of Amsterdam, Amsterdam, The Netherlands

**Keywords:** Primary immunodeficiency, primary antibody deficiency, SPAD, IgSD, prophylactic antibiotics, immunoglobulin replacement therapy, IRT, randomized controlled trial, RCT

## Abstract

**Background:**

Patients with an IgG subclass deficiency (IgSD) ± specific polysaccharide antibody deficiency (SPAD) often present with recurrent infections. Previous retrospective studies have shown that prophylactic antibiotics (PA) and immunoglobulin replacement therapy (IRT) can both be effective in preventing these infections; however, this has not been confirmed in a prospective study.

**Objective:**

To compare the efficacy of PA and IRT in a randomized crossover trial.

**Methods:**

A total of 64 patients (55 adults and 9 children) were randomized (2:2) between two treatment arms. Treatment arm A began with 12 months of PA, and treatment arm B began with 12 months of IRT. After a 3-month bridging period with cotrimoxazole, the treatment was switched to 12 months of IRT and PA, respectively. The efficacy (measured by the incidence of infections) and proportion of related adverse events in the two arms were compared.

**Results:**

The overall efficacy of the two regimens did not differ (*p* = 0.58, two-sided Wilcoxon signed-rank test). A smaller proportion of patients suffered a related adverse event while using PA (26.8% vs. 60.3%, *p* < 0.0003, chi-squared test). Patients with persistent infections while using PA suffered fewer infections per year after switching to IRT (2.63 vs. 0.64, *p* < 0.01).

**Conclusion:**

We found comparable efficacy of IRT and PA in patients with IgSD ± SPAD. Patients with persistent infections during treatment with PA had less infections after switching to IRT.

**Clinical Implication:**

Given the costs and associated side-effects of IRT, it should be reserved for patients with persistent infections despite treatment with PA.

**Electronic supplementary material:**

The online version of this article (10.1007/s10875-020-00841-3) contains supplementary material, which is available to authorized users.

## Introduction

Low or absent levels of circulating specific antibodies are the hallmark of primary antibody deficiencies (PADs), which cover a spectrum of antibody deficiency syndromes ranging from IgG subclass deficiency (IgSD) and impaired specific polysaccharide antibody production to agammaglobulinemia as the most severe antibody deficiency disease manifestation [1–3]. PADs are the most common type of primary immunodeficiency (PID) with an estimated prevalence ranging from 2.0 to 2.5/10,000 in the USA, excluding patients with IgA deficiency [4, 5]. For severe types of PAD such as common variable immunodeficiency (CVID) and X-linked agammaglobulinemia (XLA), evidence-based treatment guidelines involving immunoglobulin replacement therapy (IRT) have been developed [6–11]. In contrast, for the less severe forms of PAD such as IgSD ± specific polysaccharide antibody deficiency (SPAD), guidelines are lacking, and both prophylactic antibiotics and IRT are used. Although the costs and possible side effects of these treatments differ significantly, studies that prospectively compare these two regimens have not been published [8, 12, 13].

Patients with IgSD ± SPAD (in this study defined as patients with incomplete antibody deficiency) present with similar symptoms ranging from asymptomatic to recurrent upper and lower respiratory tract infections (URTI/LRTI) [10, 14, 15]. Although there is no general consensus regarding the treatment of these patients, multiple sources have advocated a step-up approach for the prevention of infections [8, 13, 16]. As a first-line treatment, additional vaccinations combined with increased vigilance and appropriate antibiotic therapy in the case of bacterial infections can lead to significant clinical improvement. In the absence of improvement, prophylactic antibiotics (PA) are often used to reduce the number of infections [17–19]. Patients with persistent bacterial infections despite PA can be treated with IRT to further reduce the infectious burden [16, 17, 20–23].

Open, non-placebo-controlled studies have shown that both PA and IRT can be effective in patients with IgSD and SPAD. In one study, 22 patients with an IgG2/4 deficiency and recurrent URTIs were treated with cotrimoxazole for 12 months. Twelve of the 22 patients remained symptomatic on antibiotics and were subsequently treated with IRT (intravenous immunoglobulin [IVIG], 400 mg/kg) every 3 weeks. Their mean incidence of URTIs decreased significantly after the introduction of IRT [12]. In another study, 26 patients diagnosed with chronic sinusitis and decreased serum levels of immunoglobulin isotypes, IgSD and/or SPAD were followed prospectively for 1 year on prophylactic antibiotics. Nineteen of 26 patients (74%) had a > 50% reduction in sinusitis episodes during the 12-month follow-up period [19]. A third retrospective study showed that 22 patients with IgSD and/or SPAD significantly improved after the introduction of IRT with a significant reduction in the number of infections, antibiotic use, and hospital admissions per year during the 5-year follow-up period when compared with the year prior to IRT [22]. These findings support the notion that patients with less severe forms of PAD such as IgSD ± SPAD who suffer from recurrent RTIs can benefit from either PA or IRT. Recently, a fourth retrospective study was published comparing PA (different types, *N* = 7) and IRT (*N* = 29) in children with SPAD. The authors reported a comparable mean number of infections in patients on PA vs. IRT (2.86, SD: 2.73 vs. 4.44, SD: 4.74) during the 1-year follow-up period; however, 15 patients (23.1%) failed on PA and switched to IRT during the year [24]. Moreover, the mean number of infections did not decrease in patients who received a combination of PA and IRT (*N* = 7) [24]. However, the numbers of patients in most of these studies were small, and evidence that one of these treatment modalities is truly superior to the other is lacking because these therapies have not been compared in a prospective randomized study with a larger cohort. As IRT is an expensive therapy for which global demand is increasing, it is important to establish which patients benefit the most from this type of therapy [25].

We aimed to compare the efficacy and side effects of prophylactic antibiotics vs. intravenous immunoglobulin therapy in patients with IgG subclass deficiency ± specific polysaccharide antibody deficiency using a randomized, crossover experimental design.

## Methods

### Study Design

**Fig. 1:**
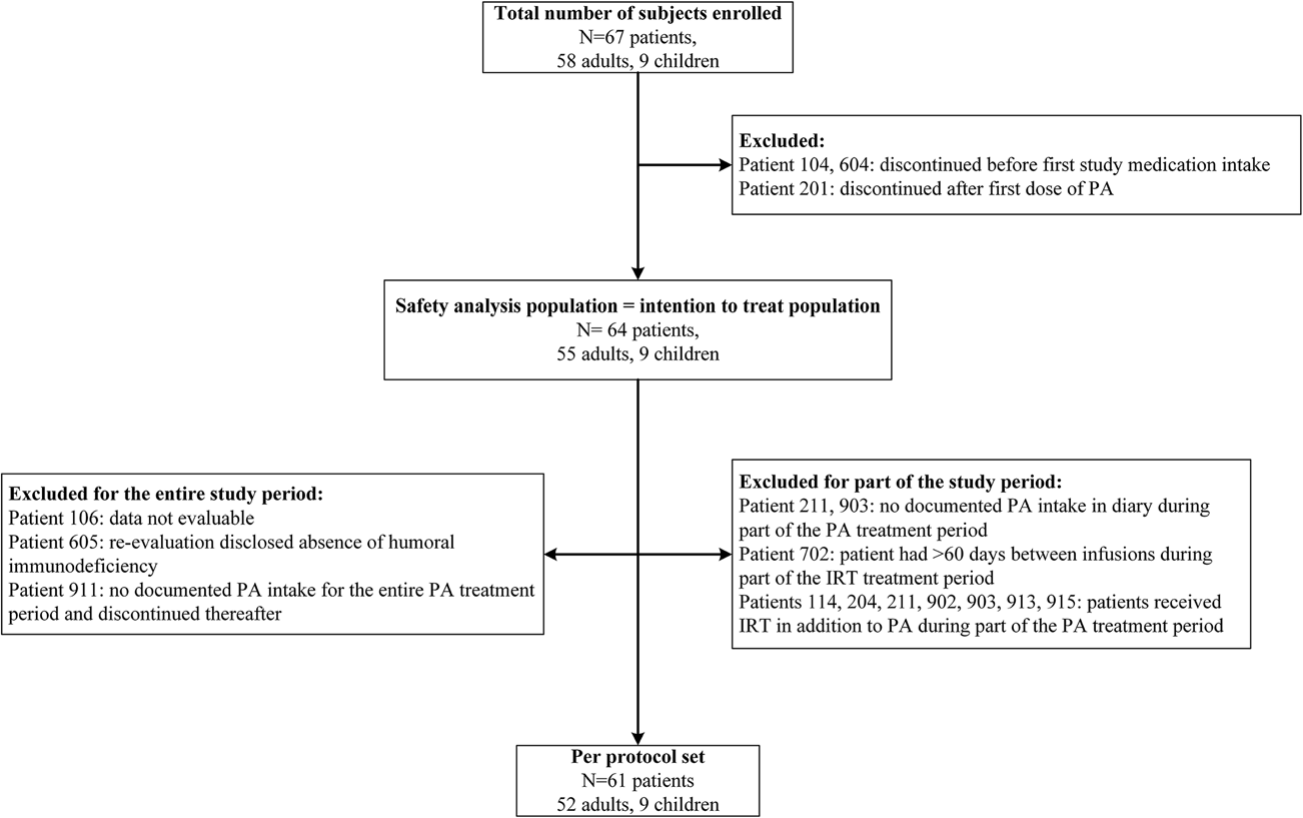
Inclusion tree of the study; 67 patients were initially enrolled of which 3 discontinued before the start of the study. A total of 55 adults and 9 children were included in the intention to treat population. For the per-protocol analysis, 52 adults and 9 children were included of which data from 8 patients was partially censored because they were treated with IVIG in addition to antibiotic prophylaxis or had no documented intake of antibiotics for part of the study period

The trial (P06.233/P08.034) was designed as a multicenter, randomized phase IV trial with a crossover design comparing the use of PA vs. IRT among IgSD and SPAD patients with recurrent infections. Block randomization was performed in a 2:2 ratio, dividing the participants between treatment arms A and B. Treatment arm A consisted of 12 months of prophylactic treatment with cotrimoxazole followed by a 3-month bridging period in which cotrimoxazole was also used as a prophylaxis, followed by 12 months of intravenous immunoglobulins (IVIG) (Fig. 1). Treatment arm B began with a 12-month period of IVIG followed by a 3-month washout period (during which antibiotics were given) and a 12-month period of prophylactic cotrimoxazole. A minimum of 45 participants was required for the assessment of the primary endpoint (the number of infections per patient per year). The Institutional Review Board (IRB) of Leiden University Medical Center operated as the central IRB that reviewed and approved this study. All participants signed written informed consent prior to participation in the study.

### Objectives and Endpoints

The primary objective was to measure the difference in the number of infections per patient per year between the two treatment modalities. The secondary predefined endpoints were a reduction in the total duration of infections, a reduction in severe infections, fewer periods of fever, fewer hospital admissions and days absent from school/work due to infections, and improvement in the Karnofsky performance skill index during the study. The number and duration of infections were reported by the treating physician and infections were classified from mild to severe using predefined definitions (Table S1).

The secondary objective was to assess side effects and tolerability through the evaluation of laboratory variables and (serious) adverse events. The intensity of adverse events (AEs) was classified as mild, moderate, or severe (Table S2). Moreover, as an exploratory objective, this study evaluated possible discriminative variables that could identify patients who benefit from IRT.

### Eligibility

This study included patients > 5 years of age with an established diagnosis of IgG_1_, IgG_2_, and/or IgG_3_ subclass and/or anti-polysaccharide antibody deficiency from eight (tertiary) hospitals in the Netherlands. IgSD was defined as IgG_1_, IgG_2_, and/or IgG_3_ serum levels below the age-adjusted lower reference range, which was determined on two occasions. SPAD was defined as an insufficient increase in anti-pneumococcal antibody formation for > 50% of the measured serotypes after vaccination with a 23-valent polysaccharide pneumococcal vaccine (Pneumo23). A sufficient increase in anti-pneumococcal antibody formation was defined as a 4-fold rise in antibody titers measured 3–5 weeks after vaccination or a serum titer > 1 mg/ml (or > 20 IU/L) after vaccination. In patients with a previous pneumococcal conjugate vaccination, only nonconjugate serotypes were considered. The diagnosis of SPAD was excluded for patients with a protective anti-pneumococcal antibody titer prior to vaccination. Other inclusion criteria were a total serum IgG level > 4 g/L and at least 2 physician-diagnosed infections in the 6 months prior to inclusion in the study.

Participants were excluded if they were treated with any other investigational drug within a week before entry into the study, if they had a history of allergic reactions to immunoglobulin treatment, if they had a progressive terminal disease or active systemic disease, if they were pregnant, or if they were known to have renal insufficiency.

### Treatment Description

IVIG was infused every 3 weeks dosed at 600 mg/kg for patients ≥ 18 years old; younger patients were given 800 mg/kg every 3 weeks. Nanogam® was used as the intravenous immunoglobulin product and was supplied by Sanquin Plasma Products BV, Amsterdam, the Netherlands [26]. Cotrimoxazole was used as an antibiotic prophylaxis, following national guidelines in the Netherlands and based on data from previous retrospective cohort studies, showing that cotrimoxazole was effective against the most prevalent bacteria causing respiratory tract infections in both PAD and non-PAD patients [12, 27–30]. Cotrimoxazole was dosed once daily at 160 mg trimethoprim/800 mg sulfamethoxazole for participants ≥ 12 years old or above 40 kg body weight, whereas younger participants were given 4 mg trimethoprim/20 mg sulfamethoxazole per kg bodyweight. If patients had a known intolerance for cotrimoxazole or if it was not tolerated during the trial, azithromycin was given 3 days per week dosed at 500 mg per day for participants > 18 years old or 10 mg per kg body weight for younger participants. Patients who developed three or more respiratory tract infections during one study period were switched to a combined regimen of IRT and PA, dosed as previously described, after the third infection.

### Laboratory Variables

As secondary markers of efficacy, we performed lymphocyte phenotyping (performed at the Medical Immunology and the Immunodiagnostic Laboratory of the Erasmus MC) [31] and mannose-binding lectin (MBL) genotyping and determined the MBL concentration (performed at the Laboratory of Blood Cell Research and Immunochemistry of Sanquin diagnostics, BV) at the start of the study. Moreover, leukocyte counts, hemoglobin levels, hematocrit, platelet counts, and potassium, alanine aminotransferase, aspartate aminotransferase, alkaline phosphatase, lactic dehydrogenase, serum creatinine, and antibody levels were measured at the start of the study and once every 3 months thereafter.

### Statistics

A minimum sample size of *N* = 35 was calculated prior to initiation of the study to prove noninferiority with a standard deviation of 2 and a noninferiority limit of 1.5 infections per year (*α* = 0.05, 90% power) between the two treatment arms. However, since a larger sample size was reached, this study was powered to prove noninferiority within a limit of 1.2 infections per patient per year between the two treatment arms. Baseline characteristics were summarized by age group, and study outcomes were summarized by treatment arm with participants classified according to the intention to treat (ITT) principle. For the mean number of infections per patient per year, the standard deviation was estimated and the difference was analyzed with a two-sided Mann-Whitney *U* test. For other endpoints, categorical outcomes were described using proportions and compared between the arms using a Chi-squared test. Outcomes were corrected for multiple comparisons using the Holms-Bonferroni approach. A Cox proportional hazard model was used to compare the time to first infection in both treatment groups. Moreover, a per-patient analysis was performed using the reduction in infection rates to define a subgroup of patients who might benefit from IRT; a reduction of at least 1 infection per year was set as the cutoff. Differences between baseline variables in patients with and without persistent infections were further visualized by fitting a hierarchal clustering model and by a supervised clustering method (partial least squares discriminant analysis, PLS-DA) using a one-component model. R Studio version 1.2.5019 and SAS version 9.4 were used to analyze the data.

## Results

### Enrolled and Randomized Participants

A total of 67 patients from 8 centers were enrolled in the study (Fig. 1) of whom 58 were adults and 9 were children. Three participants discontinued before randomization (withdrawal of informed consent), and hence, a total of 64 patients was available for the intention to treat analysis. Data from three adult participants were excluded from the per-protocol analysis for the following reasons: insufficient data (*N* = 1), misdiagnosis (no PAD, *N* = 1), and no documented intake of study medication (*N* = 1). Furthermore, data from 8 participants were partially censored for the per-protocol analysis because they were treated with IVIG in addition to antibiotic prophylaxis (*N* = 3 during IRT treatment and *N* = 5 during PA treatment) or had no documented intake of antibiotics for part of the study period.

### Baseline Characteristics

Baseline characteristics and the results of blood counts, immunoglobulin levels, B cell maturation markers, specific antibody titers, and MBL activity are presented in Table 1. After correcting for multiple comparisons, only the IgG2 levels were found to be significantly lower in children compared with those in adults (*p* < 0.0001). This was anticipated because the normal ranges for IgG2 are lower for children than for adults [32]; children and adults were pooled in further analyses. The mean IgG trough level of participants receiving IRT was 11.77 g/L (95% CI: 10.8–12.7). Mean levels of IgM and IgA did not differ significantly between children and adults included in this cohort.

**Table 1:** Baseline characteristics of the adults and children analyzed in the intention to treat analysis. Mean values were compared with a Mann-Whitney *U* test. Single asterisk indicates significance after correction for multiple comparisons using the Holms-Bonferroni method

	Adults	Children	*p* value
Sex (*n*)			
Male	21	7	
Female	34	2	
Age (median)	50	8	
Diagnosis (*n*)			
IgSD	34	2	
SPAD	3	1	
Both	18	6	
Hemoglobin (mmol/L)	8.66	8.27	0.133
Leukocytes (10^9/L)	7.80	5.06	0.023
Thrombocytes (10^9/L)	259	265	0.858
Neutrophils (10^9/L)	5.18	9.22	0.015
Eosinophils (10^9/L)	0.13	0.29	0.206
Lymphocytes (10^9/L)	2.07	2.03	0.629
Monocytes (10^9/L)	0.54	0.31	0.009
IgG			
Total IgG l	7.76	7.14	0.766
IgG1	5.12	6.05	0.143
IgG2	1.90	0.62	< 0.0001*
IgG3	0.27	0.33	0.638
IgG4	0.27	0.23	0.656
IgA	1.39	0.69	0.017
IgM	1.35	0.65	0.169
IgE (g/L)	88.7	120	0.150
CD19 (%)	11.4	11.3	0.492
CD27 (%)	33.7	17.6	0.028
CD21 (%)	85.8	88.7	0.467
CH50 (%)	107.6	102.3	0.507
AP50 (%)	94.3	66.5	0.265
MBL expression (mg/mL)	0.38	3.20	0.081
Low MBL expression (< 0.8)	21/55	2/9	
MBL polymorphism in exon 1	15/55	3/9	
MBL polymorphism in promotor	42/55	6/9	
Low MBL expression + exon 1 Polymorphism	1/55	0/9	
Low MBL expression + promoter polymorphism	2/55	0/9	
Low MBL expression + exon 1 and promoter polymorphisms	13/55	2/9	

*IgSD* immunoglobulin G subclass deficiency, *SPAD* specific polysaccharide antibody deficiency, *Ig* immunoglobulin, *CD* cluster of differentiation, and *MBL* mannose-binding lectin

### Primary Outcome: Infection Prevention

**Fig. 2:**
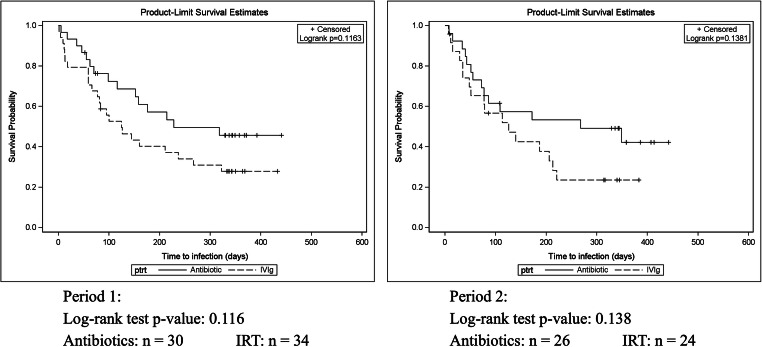
Kaplan-Meier curves for the time to first infection for treatment period 1 (left) and treatment period 2 (right). There was a trend towards a longer time to the first infection for the antibiotics group, but this was not statistically significant (*p* = 0.116 and *p* = 0.138, respectively, for periods 1 and 2)

A total of 64 participants were evaluable for the primary endpoint analysis (IRT *N* = 58, PA *N* = 56). A total of 19,618 treatment days were recorded for the IRT group vs. 20,256 for the PA group. There was no proof of statistically significant superiority of either treatment arm with a mean of 1.76 (SD: 1.92) infections per patient per year in the IRT arm vs. 1.55 (SD: 1.94) in the PA arm (Table 2). In the IRT group, 76.4% of the infections were RTIs vs. 73.8% of those in the PA group. Of all the participants, 70.7% in the IRT arm and 51.8% in the PA arm had at least one infection during the intervention period. However, this was not found to be significant in a Cox proportional hazards model, although a trend towards a longer infection free survival during PA treatment could be identified in both treatment periods (*p* = 0.116 and *p* = 0.138, Fig. 2). To evaluate the therapeutic potential of a combined therapy with both IRT and PA, the censored data of the 7 participants receiving a combination therapy was compared with the study population; however, no significant reduction in the incidence of infection was found.

**Table 2:** Primary outcome measures in the intention to treat (ITT) and the per-protocol set (PPS). Chi-squared tests or Mann-Whitney *U* tests were used accordingly to calculate the *p* values; no significant differences were found

		ITT			PPS		
		IRT	PA	*p* value	IRT	PA	*p* value
Number of patients	All	58	56		57	54	
	Children	7	9		7	9	
	Adults	51	47		50	45	
Number of treatment days	Sum	19,618	20,256		19,228	19,495	
	Mean (SD)	338.2 (85.3)	361.7 (79.9)		337.3 (90.5)	361.0 (73.8)	
Number of infections	All	89	84		88	82	
	Children	4	10		4	10	
	Adults	85	74		84	72	
Duration (*n*)	Acute	84	75		83	73	
	Chronic	5	9		5	9	
Intensity (*n*)	Mild	48	49		48	47	
	Moderate	35	30		34	30	
	Severe	6	5		6	5	
Patients suffering ≥ 1 infection (%)		41 (70.7%)	29 (51.8%)	0.09	40 (70.2%)	29 (53.7%)	0.15
Infections per patient per year (SD)		1.76 (1.92)	1.55 (1.94)	0.56	1.77 (1.94)	1.57 (1.90)	0.58
Severe infections per patient per year (SD)		0.11 (0.60)	0.09 (0.36)	0.83	0.11 (0.60)	0.09 (0.36)	0.83
Days of infection per patient year (SD)		24.6 (30.0)	23.1 (42.0)	0.83	24.6 (30.3)	23.7 (42.4)	0.85

*ITT* intention to treat, *PPS* per-protocol set, *IRT* immunoglobulin replacement therapy, *PA* prophylactic antibiotics, and *SD* standard deviation

### Secondary Outcome Parameters

To further quantify the effect of the treatments in both study arms, secondary outcome measures were compared. The mean total duration of infections per year and the number of severe infections (Table 2), days off school/work, febrile episodes, and hospitalization admissions were analyzed (Table 3); no significant differences were found. The mean total duration of infections per patient per year was 24.6 days (SD: 30.0 days) in the IRT group vs. 23.1 days (SD: 42.0 days) in the PA group. The mean number of severe infections per patient per year was 0.11 (SD: 0.60) in the IRT group vs. 0.09 (SD: 0.36) in the PA group. Respiratory tract infections were the most commonly reported infection (68% of all infections) and were equally reported in both treatment arms.

**Table 3:** Secondary outcome measures in the intention to treat (ITT) and the per-protocol set (PPS). Chi-squared tests or Mann-Whitney *U* tests were used accordingly to calculate *p* values

	ITT			PPS		
	IRT	PA	*p* value	IRT	PA	*p* value
Total number of patients	58	56		57	54	
Total number of infections	89	84		88	82	
Days off work or school						
Total number of days off work/school	120	65		90	65	
Patients (%) with at least one day off work/school	7 (12.1%)	7 (12.5%)	0.94	6 (10.5%)	7 (13.0%)	0.61
Number of days off work/school per patient per year (mean ± SD)	2.3 (9.3)	1.2 (3.4)	0.41	1.6 (7.9)	1.2 (3.4)	0.73
Fever						
Total number of fever events	21	24		21	22	
Patients (%) experiencing at least one fever event	12 (20.7%)	13 (23.2%)	0.71	12 (21.1%)	13 (24.1%)	0.66
Number of fever events per patient per year (mean ± SD)	0.4 (1.2)	0.5 (1.0)	0.63	0.5 (1.2)	0.4 (1.0)	0.64
Hospitalization due to infection						
Total number of hospital admissions due to infection	14	10		13	10	
Total number of days in the hospital due to infection	112	81		89	81	
Patients (%) with at least one hospital admission	6 (10.3%)	7 (12.5%)	0.65	5 (8.8%)	7 (13.0%)	0.37
Number of hospital admissions per patient per year	0.30 (1.10)	0.19 (0.58)	0.51	0.28 (1.10)	0.20 (0.59)	0.64
Average number of days in the hospital per person year (mean ± SD)	2.80 (10.11)	1.49 (4.22)	0.37	2.32 (9.51)	1.55 (4.29)	0.59

*ITT* intention to treat, *PPS* per protocol set, *IRT* immunoglobulin replacement therapy, and *PA* prophylactic antibiotics

### Predictors of Benefit from IRT

To evaluate the possibility of a subgroup of patients who benefited from IRT over PA, a per-patient analysis was performed. The reported infection rates were analyzed and the reductions in infection rates were calculated. In this analysis, we identified 11/58 patients with a reduction of at least 1 infection per year when treated with IRT instead of antibiotics (monotherapy, as prescribed in the treatment protocol). To further characterize these participants, a PLS-DA was performed on all baseline characteristics available. This analysis yielded no markers that could identify this subgroup of patients who benefited from IRT over PA. Next, we analyzed the number of infections during treatment with PA as a separate variable, and found that patients who suffered ≥ 2 infections during treatment with PA generally had a beneficial response when switched to IRT. Specifically, the occurrence of ≥ 2 infections despite the use of PA monotherapy identified participants who subsequently improved following a switch to IRT monotherapy with 80% (CI: 44.4–97.5) sensitivity and 80.6% specificity (CI: 64.0–91.8). Moreover, most of these infections (86%) were RTIs. In this subgroup of patients with persistent infections despite PA, there was a significant (*p* < 0.01) reduction in the mean number of RTIs from 2.64 (SD: 2.20) RTIs per patient per year to 0.64 (SD: 0.81) for patients treated with IRT. Finally, we analyzed whether we could identify characteristics that could distinguish patients who had a reduction of at least 1 infection when treated with PA instead of IRT (28/58), but none could be found. Moreover, we found an absolute reduction in the mean number of airway infections per year in those who benefited from PA over IRT of 1.03, versus 2.0 in the patients who benefited from IRT over PA (*p* = 0.051).

### Tolerability of Medication in Both Treatment Arms

The key secondary objective of this study was to compare the tolerability of PA and IRT by analyzing the proportion of patients who suffered any adverse events (AE) that were possibly related to treatment. A total of 64 participants received at least one dose of study medication and had reports of safety measurements. Overall, 37 subjects (66.1%) experienced at least one AE during PA treatment, while there were only 46 such subjects (79.3%) during IRT. The most commonly reported AEs for both treatment arms were diarrhea, nausea, fatigue, pyrexia, headache, and rash for both the total number of AEs and the AEs possibly related to treatment (Table S3). In total, 40 participants (62.5%) experienced at least one AE that was at least potentially related to the study drug: 15 in the PA arm (26.8%) and 35 in the IRT arm (60.3%, *p* = 0.0003, Table 4). Patients treated with IRT experienced headache more often than patients treated with PA (36.2% vs. 1.8%, *p* < 0.0001). The intensity of the adverse events that were possibly related to treatment was lower in the PA arm than in the IRT arm; 25% of patients in the PA arm had AEs of mild intensity, 1.8% of patients had AEs of moderate intensity, and 0% of patients had AEs of severe intensity vs. 53.4%, 19.0%, and 3.4% in the IRT arm, respectively (*p* = 0.002). Most (60%) of the AEs reported in the IRT group were reported during the first four infusions. Moreover, 8 patients (5 in treatment arm a, 3 in treatment arm B) were treated with azithromycin, due to known or acquired intolerability of cotrimoxazole.

**Table 4:** Total numbers and proportions of (serious) adverse events among the patients in the antibiotics and IRT groups. Chi-squared tests were used to calculate the *p* values. Single asterisk indicates significance after correction for multiple comparisons using the Holms-Bonferroni method. The total number of events and the number of related serious events were lower in the antibiotics group

	Statistic	PA	IRT	*p* value
Any adverse events	Number of events % (*n*/*N*)	93 66.1% (37/56)	270 79.3% (46/58)	0.1149
Any related adverse events	Number of events % (*n*/*N*)	34 26.8% (15/56)	184 60.3% (35/58)	0.0003*
Any serious adverse events (total)	Number of events % (*n*/*N*)	13 12.5% (7/56)	26 19.0% (11/58)	0.3437
Serious adverse events (AE-related)	Number of events % (*n*/*N*)	1 1.8 (1/56)	14 13.8% (8/58)	0.0181*
Serious adverse event (infection-related)	Number of events % (*n*/*N*)	12 12.5% (7/56)	12 10.3% (6/58)	0.7128

*PA* prophylactic antibiotics, *IRT* immunoglobulin replacement therapy, and *AE* adverse event

Overall, 13 serious adverse events (SAE) were reported by 7 participants (12.5%) during PA and 26 SAEs were reported by 11 participants (19%) during IRT. Of these SAEs, 24 originated from infection-related events (*N* = 12 during PA and *N* = 12 during IRT). One of the SAEs was identified as probably related to IRT. The patient was admitted to the hospital with fever, lymphadenopathy, myalgia, arthralgia, malaise, and leucopenia of unknown origin from which the patient recovered after 36 days, during which IRT was continued.

## Discussion

This multicenter, randomized, controlled crossover trial is the first to analyze the efficacy of PA versus IRT in patients with IgSD ± SPAD. Overall, our data did not demonstrate a significant difference in infection-related parameters between the two regimens, and PA was better tolerated than IRT. However, the subgroup of patients with persistent infections despite treatment with PA had significantly fewer infections when treated with IRT.

Overall, the data from this study are generalizable to other patients with IgSD ± SPAD and recurrent infections, as trial eligibility criteria allowed the participation by both children and adults with IgSD, SPAD, or both, and children and adults from both disease categories were included. Only four patients were included that suffered specifically from SPAD, making this study less generalizable for that patient group. However, the mean infection rates reported were comparable with those reported in two earlier studies, one in children with SPAD [24] and one in adults with IgSD and SPAD [22]. The current study design, a randomized, controlled crossover trial, was selected as the optimal design to show differences in the efficacy of prophylactic treatment in a relatively rare disease where the cohort sizes are expected to be small [33]. Both treatment arms covered 12 months to avoid seasonal differences in infections. To ensure that successive infections were equally weighted in the analysis, the duration and severity of infections were included in the analyses. Moreover, good compliance was achieved as 53/64 patients completely adhered to the protocol, resulting in sample sizes sufficient to perform a per-protocol analysis.

The crossover design of the study allowed further study of the potential benefit of IRT over PA. Using subgroup analysis, we were able to define a subgroup of patients with persistent infections despite PA treatment who benefited from switching to IRT. This group had ≥ 2 infections per year despite treatment with PA and showed a statistically significant (*p* < 0.01) and clinically relevant reduction in infections after switching to IRT. Apart from the persistence of infections while using PA, we found no other (laboratory) parameters identifying patients who would benefit from IRT over PA. Joud Hajjar et al. showed that patients with persistent infections had lower IgG titers than patients who were responsive to PA or IRT; however, we could not replicate this result in our cohort [24]. We also analyzed MBL, as MBL deficiency was hypothesized to be only clinically relevant in patients with concurrent immune deficiencies; however, we found no clinically relevant effect of MBL deficiency on infections even in combination with the antibody deficiencies studied in our protocol. Our data suggest that an empirical step-up approach, beginning with antibiotic prophylaxis and switching to IRT monotherapy in response to persistent infections, is valid for the treatment of IgSD ± SPAD. IRT reduced the infectious burden in patients on PA who suffered from two or more infections in 56% of the cases.

The tolerability analyses of this study showed that more AEs that were possibly related to treatment occurred in the IRT group. However, the majority of these AEs occurred during the initial IRT infusions, and most of these AEs were transient, mild, and were well-known adverse reactions to IVIG. It is known that the number of AEs decreases over time, as dosage and infusion rate are adjusted based on their occurrence [34]. Moreover, the risk of AEs differed in the two study arms, as patients with a known intolerability to cotrimoxazole or who experienced side effects from antibiotics during the study were administered an alternative drug (azithromycin instead of cotrimoxazole), whereas the protocol for patients on IRT did not include an option for a different type of IRT in the study. Overall, 8 patients were treated with azithromycin instead of cotrimoxazole, which may have caused a reduction in AEs within the PA group.

The limitations of this study are similar to those of other smaller randomized studies of rare diseases, which are often constrained by recruitment challenges. First, during recruitment, several study candidates chose not to participate in the study, as they preferred to adhere to the treatment they currently received. It is not clear how many patients received no treatment, PA, or IRT, and whether this may have introduced selection bias. However, the study still had sufficient power to show that there was no significant difference in the efficacy between PA and IRT and was able to detect noninferiority with a limit of 1.2 infections per patient per year. The study was not sufficiently powered to detect a difference in the infection free survival time between the two study arms, or to draw any concrete conclusions for patients affected by SPAD specifically. Second, the protocol described relatively high substitution doses of IVIG, which were not always achieved. However, it is known that the dose of IVIG is not a good measure of effectivity and that IgG trough levels in combination with a reduced frequency of sinopulmonary infections are recommended instead [9, 11]. In CVID, trough levels of 6–8 g/L have been advised for the optimal prevention of infections; this was achieved in all patients who received IRT in this study [35, 36]. Third, this study did not analyze the effect of PA on antibiotic resistance. However, a recent systematic review that studied the use of PA in patients with chronic obstructive pulmonary disease found no direct evidence for an increase in antibiotic resistance [37]. A recent study in CVID patients confirmed this for azithromycin in PID patients in Italy during a 3-year follow-up period [38]. However, these findings might not be generalizable to other countries, since antibiotic resistance is a multifactorial issue depending, among others, on local resistance patterns and the extent of local prescription protocols. Antibiotic resistance is a growing problem that could represent a serious threat particularly in immunodeficient patients who are more susceptible to infections [39, 40]. Finally, the results of this study might not be replicable in a non-trial setting due to a possible difference in patient compliance between the two regimens. Non-compliance for oral medication has been extensively studied in the past and up to 50% of patients have been reported as non-compliant with (oral) medication [41]. Due to the nature of administration regimens of IRT, involving day-care settings or home administration requiring specialized personnel, being treated with IRT, could counteract some of the hurdles patients experience in complying to prophylactic treatment [42].

In conclusion, we found that overall, prophylactic antibiotics and IRT were equally efficient in preventing infections in a cohort of patients with IgSD ± SPAD. However, a subgroup of patients with persistent infections during treatment with PA showed a significant reduction in infections after switching to IRT. Future research should focus on identifying biomarkers to better define this group.

## Electronic Supplementary Material

ESM 1 (DOCX 41 kb).

## Abbreviations

CVID: Common variable immunodeficiency
IgSD: IgG subclass deficiency
IRT: Immunoglobulin replacement therapy
IVIG: Intravenous immunoglobulins
MBL: Mannose-binding lectin
PA: Prophylactic antibiotics
PAD: Primary antibody deficiency
PID: primary immunodeficiencies
PLS-DA: Partial least squares discriminant analysis
SPAD: Specific polysaccharide antibody deficiency
URTI/LRTI: Upper/lower respiratory tract infections
XLA: X-linked agammaglobulinemia
