# Supplementary material for: Immunoglobulin Replacement Therapy Versus Antibiotic Prophylaxis as Treatment for Incomplete Primary Antibody Deficiency
Source: J Clin Immunol. 2020 Nov 18;41(2):382–92. doi: 10.1007/s10875-020-00841-3 (PMC7858555; doi:10.1007/s10875-020-00841-3)
Supplement: Supplementary file 1 — (DOCX 41 kb). [file 10875_2020_841_MOESM1_ESM.docx]

**Supplemental Tables**

**Table S1. Examples of Infections Classified into Mild, Moderate and Severe^a^**

| **Mild** | **Moderate**^a^ | **Severe**^a^ |
| --- | --- | --- |
| Nasal discharge | Sinusitis^b^ | Pneumonia |
| Flu-like illness | Bronchitis | Pleurisy/pleuritis |
| Conjunctivitis | Pharyngitis | Acute sinusitis^b^ |
| Diarrhoea | Laryngitis | Acute abscesses |
| Vaginal infection | Otitis | Sepsis |
| Oral Candida | Fever^c^ | Acute Osteomyelitis |
| Afebrile enteritis | Urinary tract infection | Meningitis |

^a^ Moderate infections did not require bed rest or absence from school or work.

Severe infections always required bed rest or hospitalisation and absence from school or work.

^b^ Moderate sinus-pressure, headaches
 Severe sinusitis-with fever, positive x-rays and necessity of antibiotics

^c^ Fever of unknown origin lasting no more than 3 days

**Table S2. Adverse Event intensity and severity grading criteria**

| **Adverse Event grading criteria** |  |
| --- | --- |
| **Intensity** | **Seriousness** |
| **Mild:** The patient is aware of the sign/symptom, but it does not interfere with his/her usual activities and/or is of no clinical consequence. | **Severe (SAE**): A serious adverse event is any untoward medical occurrence or effect that at any dose:  - results in death or is life threatening;  - requires hospitalisation or prolongation of hospitalisation;  - results in persistent or significant disability or incapacity;  - is a congenital anomaly or birth defect;  - is a new event of the trial likely to affect the safety of the subjects |
| **Moderate:** The adverse event interferes with usual activities of the patient, or is of some clinical consequence. | **Non-serious:** All events that cannot be graded as serious. |
| **Severe:** The patient is unable to carry out his/her usual activities and if applicable unable to go to school, or the adverse event is of definite clinical consequence. |  |

**Table S3. Treatment Emergent Adverse Events: Incidence by System Organ Class and Preferred Term**

|  | **Antibiotic Period 1+2** | | | | **IVIg Period 1+2** | |
| --- | --- | --- | --- | --- | --- | --- |
| **System Organ Class Preferred Term** | **Events** | | | **%(n/N)** | **Events** | **%(n/N)** |
| OVERALL | 93 | | | 66.1% (37/56) | 270 | 79.3% (46/58) |
| Blood And Lymphatic System Disorders | 0 | | | - | 3 | 5.2% (3/58) |
| Anaemia | 0 | | | - | 2 | 3.4% (2/58) |
| Leukopenia | 0 | | | - | 1 | 1.7% (1/58) |
| Cardiac Disorders | 0 | | | - | 2 | 3.4% (2/58) |
| Palpitations | 0 | | | - | 2 | 3.4% (2/58) |
| Ear And Labyrinth Disorders | 2 | | | 3.6% (2/56) | 0 | - |
| Cerumen Impaction | 1 | | | 1.8% (1/56) | 0 | - |
| Tinnitus | 1 | | | 1.8% (1/56) | 0 | - |
| Eye Disorders | 1 | | | 1.8% (1/56) | 0 | - |
| Cataract | 1 | | | 1.8% (1/56) | 0 | - |
| Gastrointestinal Disorders | 27 | | | 26.8% (15/56) | 28 | 24.1% (14/58) |
| Abdominal Discomfort | 1 | | | 1.8% (1/56) | 1 | 1.7% (1/58) |
| Abdominal Pain | 3 | | | 5.4% (3/56) | 2 | 3.4% (2/58) |
| Abdominal Pain Upper | 2 | | | 3.6% (2/56) | 0 | - |
| Constipation | 2 | | | 3.6% (2/56) | 1 | 1.7% (1/58) |
| Diarrhoea | 8 | | | 10.7% (6/56) | 8 | 6.9% (4/58) |
| Diverticulum | 0 | | | - | 1 | 1.7% (1/58) |
| Dyspepsia | 2 | | | 3.6% (2/56) | 0 | - |
| Gastric Disorder | 1 | | | 1.8% (1/56) | 0 | - |
| Gastrointestinal Motility Disorder | 1 | | | 1.8% (1/56) | 1 | 1.7% (1/58) |
| Haematochezia | 0 | | | - | 1 | 1.7% (1/58) |
| Haemorrhoids | 0 | | | - | 1 | 1.7% (1/58) |
| Nausea | 6 | | | 8.9% (5/56) | 11 | 12.1% (7/58) |
| Oesophagitis | 1 | | | 1.8% (1/56) | 0 | - |
| Vomiting | 0 | | | - | 1 | 1.7% (1/58) |
| General Disorders And Administration Site Conditions | 11 | | | 8.9% (5/56) | 76 | 48.3% (28/58) |
| Asthenia | 0 | | | - | 1 | 1.7% (1/58) |
| Chest Discomfort | 0 | | | - | 1 | 1.7% (1/58) |
| Chest Pain | 0 | | | - | 3 | 5.2% (3/58) |
| Chills | 0 | | | - | 5 | 5.2% (3/58) |
| Discomfort | 0 | | | - | 5 | 3.4% (2/58) |
| Fatigue | 4 | | | 5.4% (3/56) | 15 | 10.3% (6/58) |
| Feeling Cold | 0 | | | - | 5 | 3.4% (2/58) |
| Gait Disturbance | 0 | | | - | 1 | 1.7% (1/58) |
| Influenza Like Illness | 1 | | | 1.8% (1/56) | 4 | 5.2% (3/58) |
| Infusion Site Erythema | 0 | | | - | 2 | 3.4% (2/58) |
| Infusion Site Extravasation | 0 | | | - | 2 | 1.7% (1/58) |
| Infusion Site Inflammation | 0 | | | - | 1 | 1.7% (1/58) |
| Infusion Site Phlebitis | 0 | | | - | 3 | 1.7% (1/58) |
| Infusion Site Rash | 0 | | | - | 1 | 1.7% (1/58) |
| Malaise | 0 | | | - | 4 | 6.9% (4/58) |
| Oedema Peripheral | 0 | | | - | 1 | 1.7% (1/58) |
| Pyrexia | 6 | | | 5.4% (3/56) | 22 | 15.5% (9/58) |
| Immune System Disorders | 2 | | | 3.6% (2/56) | 1 | 1.7% (1/58) |
| Drug Hypersensitivity | 1 | | | 1.8% (1/56) | 0 | - |
| Seasonal Allergy | 1 | | | 1.8% (1/56) | 1 | 1.7% (1/58) |
| Infections And Infestations | 7 | | | 10.7% (6/56) | 8 | 10.3% (6/58) |
| Body Tinea | 1 | | | 1.8% (1/56) | 0 | - |
| Folliculitis | 2 | | | 1.8% (1/56) | 0 | - |
| Fungal Skin Infection | 0 | | | - | 2 | 3.4% (2/58) |
| Herpes Simplex | 0 | | | - | 1 | 1.7% (1/58) |
| Lymphangitis | 0 | | | - | 1 | 1.7% (1/58) |
| Nasopharyngitis | 1 | | | 1.8% (1/56) | 4 | 6.9% (4/58) |
| Rhinitis | 1 | | | 1.8% (1/56) | 0 | - |
| Vulvovaginal Mycotic Infection | 2 | | | 3.6% (2/56) | 0 | - |
| Injury, Poisoning And Procedural Complications | 4 | | | 5.4% (3/56) | 7 | 8.6% (5/58) |
| Contusion | 0 | | | - | 1 | 1.7% (1/58) |
| Corneal Abrasion | 1 | | | 1.8% (1/56) | 0 | - |
| Fall | 1 | | | 1.8% (1/56) | 1 | 1.7% (1/58) |
| Hand Fracture | 0 | | | - | 1 | 1.7% (1/58) |
| Incorrect Route Of Drug Administration | 0 | | | - | 1 | 1.7% (1/58) |
| Joint Dislocation | 0 | | | - | 1 | 1.7% (1/58) |
| Joint Injury | 0 | | | - | 1 | 1.7% (1/58) |
| Limb Injury | 0 | | | - | 1 | 1.7% (1/58) |
| Post Procedural Haematoma | 1 | | | 1.8% (1/56) | 0 | - |
| Rib Fracture | 1 | | | 1.8% (1/56) | 0 | - |
| Investigations | 3 | | | 5.4% (3/56) | 3 | 3.4% (2/58) |
| Alanine Aminotransferase Increased | 0 | | | - | 1 | 1.7% (1/58) |
| Aspartate Aminotransferase Increased | 0 | | | - | 1 | 1.7% (1/58) |
| Biopsy Prostate | 1 | | | 1.8% (1/56) | 0 | - |
| Blood Creatinine Increased | 2 | | | 3.6% (2/56) | 0 | - |
| Laboratory Test Abnormal | 0 | | | - | 1 | 1.7% (1/58) |
| Metabolism And Nutrition Disorders | 2 | | | 3.6% (2/56) | 0 | - |
| Hypercholesterolaemia | 1 | | | 1.8% (1/56) | 0 | - |
| Hyperkalaemia | 1 | | | 1.8% (1/56) | 0 | - |
| Musculoskeletal And Connective Tissue Disorders | 9 | | | 12.5% (7/56) | 17 | 22.4% (13/58) |
| Arthralgia | 2 | | | 1.8% (1/56) | 3 | 3.4% (2/58) |
| Arthritis | 0 | | | - | 1 | 1.7% (1/58) |
| Arthropathy | 0 | | | - | 1 | 1.7% (1/58) |
| Back Pain | 2 | | | 3.6% (2/56) | 2 | 3.4% (2/58) |
| Bursitis | 0 | | | - | 1 | 1.7% (1/58) |
| Intervertebral Disc Protrusion | 0 | | | - | 1 | 1.7% (1/58) |
| Muscle Spasms | 1 | | | 1.8% (1/56) | 0 | - |
| Musculoskeletal Pain | 1 | | | 1.8% (1/56) | 0 | - |
| Musculoskeletal Stiffness | 0 | | | - | 1 | 1.7% (1/58) |
| Myalgia | 1 | | | 1.8% (1/56) | 3 | 5.2% (3/58) |
| Osteitis | 0 | | | - | 1 | 1.7% (1/58) |
| Osteoporosis | 1 | | | 1.8% (1/56) | 0 | - |
| Pain In Extremity | 0 | | | - | 1 | 1.7% (1/58) |
| Pain In Jaw | 0 | | | - | 1 | 1.7% (1/58) |
| Systemic Lupus Erythematosus | 1 | | | 1.8% (1/56) | 0 | - |
| Tendonitis | 0 | | | - | 1 | 1.7% (1/58) |
| Neoplasms Benign, Malignant And Unspecified (Incl Cysts And Polyps) | 2 | | | 3.6% (2/56) | 1 | 1.7% (1/58) |
| Basal Cell Carcinoma | 1 | | | 1.8% (1/56) | 0 | - |
| Gastrointestinal Tract Adenoma | 0 | | | - | 1 | 1.7% (1/58) |
| Lung Neoplasm | 1 | | | 1.8% (1/56) | 0 | - |
| Nervous System Disorders | 11 | | | 14.3% (8/56) | 71 | 48.3% (28/58) |
| Aphasia | 0 | | | - | 1 | 1.7% (1/58) |
| Disturbance In Attention | 0 | | | - | 1 | 1.7% (1/58) |
| Dizziness | 0 | | | - | 1 | 1.7% (1/58) |
| Essential Tremor | 0 | | | - | 1 | 1.7% (1/58) |
| Headache | 8 | | | 12.5% (7/56) | 60 | 39.7% (23/58) |
| Migraine | 0 | | | - | 2 | 1.7% (1/58) |
| Sciatica | 1 | | | 1.8% (1/56) | 0 | - |
| Sensory Disturbance | 0 | | | - | 1 | 1.7% (1/58) |
| Sinus Headache | 2 | | | 1.8% (1/56) | 3 | 3.4% (2/58) |
| Syncope | 0 | | | - | 1 | 1.7% (1/58) |
| Psychiatric Disorders | 1 | | | 1.8% (1/56) | 2 | 3.4% (2/58) |
| Attention Deficit/Hyperactivity Disorder | 1 | | | 1.8% (1/56) | 1 | 1.7% (1/58) |
| Depression | 0 | | | - | 1 | 1.7% (1/58) |
| Renal And Urinary Disorders | 0 | | | - | 2 | 3.4% (2/58) |
| Nephrolithiasis | 0 | | | - | 2 | 3.4% (2/58) |
| Reproductive System And Breast Disorders | 1 | | | 1.8% (1/56) | 0 | - |
| Fibrocystic Breast Disease | 1 | | | 1.8% (1/56) | 0 | - |
| Respiratory, Thoracic And Mediastinal Disorders | 4 | | | 7.1% (4/56) | 17 | 19.0% (11/58) |
| Asthma | 0 | | | - | 2 | 3.4% (2/58) |
| Chronic Obstructive Pulmonary Disease | 1 | | | 1.8% (1/56) | 5 | 3.4% (2/58) |
| Cough | 0 | | | - | 1 | 1.7% (1/58) |
| Diaphragmatic Hernia | 1 | | | 1.8% (1/56) | 0 | - |
| Dyspnoea | 1 | | | 1.8% (1/56) | 5 | 6.9% (4/58) |
| Nasal Discomfort | 0 | | | - | 1 | 1.7% (1/58) |
| Nasal Obstruction | 1 | | | 1.8% (1/56) | 1 | 1.7% (1/58) |
| Rhinitis Allergic | 0 | | | - | 1 | 1.7% (1/58) |
| Rhinorrhoea | 0 | | | - | 1 | 1.7% (1/58) |
| Skin And Subcutaneous Tissue Disorders | 3 | | | 3.6% (2/56) | 26 | 24.1% (14/58) |
| Angioedema | 1 | | | 1.8% (1/56) | 0 | - |
| Blister | 0 | | | - | 2 | 1.7% (1/58) |
| Dyshidrosis | 0 | | | - | 1 | 1.7% (1/58) |
| Eczema | 0 | | | - | 1 | 1.7% (1/58) |
| Exfoliative Rash | 0 | | | - | 1 | 1.7% (1/58) |
| Hyperhidrosis | 0 | | | - | 1 | 1.7% (1/58) |
| Ingrowing Nail | 0 | | | - | 1 | 1.7% (1/58) |
| Night Sweats | 0 | | | - | 1 | 1.7% (1/58) |
| Pruritus | 0 | | | - | 2 | 3.4% (2/58) |
| Psoriasis | 1 | | | 1.8% (1/56) | 0 | - |
| Rash | 0 | | | - | 12 | 10.3% (6/58) |
| Rash Pruritic | 0 | | | - | 2 | 1.7% (1/58) |
| Skin Depigmentation | 0 | | | - | 1 | 1.7% (1/58) |
| Skin Lesion | 0 | | | - | 1 | 1.7% (1/58) |
| Urticaria | 1 | | | 1.8% (1/56) | 0 | - |
| Surgical And Medical Procedures | 2 | | | 3.6% (2/56) | 1 | 1.7% (1/58) |
| Central Venous Catheterisation | 0 | | | - | 1 | 1.7% (1/58) |
| Intra-Uterine Contraceptive Device Insertion | 1 | | | 1.8% (1/56) | 0 | - |
| Mammoplasty | 1 | | | 1.8% (1/56) | 0 | - |
| Vascular Disorders | 1 | | | 1.8% (1/56) | 5 | 6.9% (4/58) |
| Hot Flush | 0 | | | - | 1 | 1.7% (1/58) |
| Hypertension | 1 | | | 1.8% (1/56) | 2 | 1.7% (1/58) |
| Phlebitis | 0 | | | - | 2 | 3.4% (2/58) |
| Source: Table 14.3.01.04 | |  |  |  |  |  |
| MedDRA version 14.1 was used | | |  |  |  |  |
